# Investigation of authors’ self-citation in contemporary forensic odontology literature

**DOI:** 10.1007/s12024-024-00928-y

**Published:** 2024-12-11

**Authors:** N. Angelakopoulos, N. Polukhin, S.B. Balla

**Affiliations:** 1 Department of Orthodontics and Dentofacial Orthopedics, University of Bern, Bern, 3010 Switzerland; 2 Department of Public Health and Medical Social Sciences, Synergy University, Moscow, Russian Federation; 3 La Trobe Rural Health School, La Trobe University, Bendigo, Australia

**Keywords:** Bibliometrics, Forensic odontology, Forensic science journals, Author self-citation

## Abstract

This bibliometric investigation aimed to analyze trends in author self-citation within prominent forensic odontology literature and explore potential correlations between self-citation rates and publication attributes. We reviewed seven leading forensic sciences journals from 2003 to 2023. For this analysis, we focused on two specific timeframes: 2003–2007 and 2018–2023. Our review encompassed original research articles, reviews, and case reports. Eligible articles underwent detailed examination for article and author attributes and citation metrics. Utilizing univariable and multivariable negative binomial regression analyses, we explored potential associations between the number of self-citations and various publication characteristics. This study analyzed 415 articles related to forensic odontology, of which 237 (57.1%) included at least one self-citation. Key findings highlight prevalent topics such as dental age estimation and human dental identification. A significant portion of the studies involved prospective, retrospective, and cross-sectional designs, and there has been a notable increase in the number of reviews and meta-analyses in recent years compared to an equivalent past period. Self-citation was observed in over half of the analyzed articles, with a median total citation count of 31 and a median self-citation rate of 7.5%. Further bibliometric investigation is required to establish definitive conclusions regarding author self-citation patterns in forensic odontology literature, particularly by exploring longer time spans.

## Introduction

Forensic odontology (FO) is a niche science that stands at the intersection of dentistry and law and plays an important role in investigating various legal cases involving dental evidence. FO, also known as forensic dentistry and forensic odontostomatology, was defined by Keiser-Nielson [1] as the branch of forensic medicine dealing with the examination and evaluation of dental evidence. As with any scientific field, the integrity and reliability of research within FO rely heavily on the ethical conduct of its practitioners and scholars. One aspect that has garnered attention in recent years is the phenomenon of author self-citation, a practice wherein authors cite their previous work within their publications [2]. While self-citation can be a legitimate and valuable scholarly practice, its extent and patterns within the literature have raised questions regarding potential implications for research quality, objectivity, and academic integrity [3]. The investigation of author self-citation within FO has never been explored, and the small one-on-one discussions in the academic community regarding the nature, motivations, and consequences of self-citation have never been proven or published. Lawani (1982) [4] delved into the heterogeneity and classification of author self-citations, shedding light on the various forms this practice can take. Ioannidis (2015) [5] provided a generalized view of self-citation, distinguishing between direct, co-author, collaborative, and coercive-induced self-citation and highlighting the complexities involved in its analysis.

In contemporary academia, the productivity of scholars significantly influences the ranking of institutions, career advancement in academia, and funding allocation [6]. To assess the impact and significance of individual researchers’ scientific contributions, metrics such as citation counts and the h-index are routinely evaluated by tenure-track committees and grant advisory boards. Essentially, an author’s h-index reflects the number of publications garnered at least the same number of citations, including self-citations referencing their prior work [7]. Self-citation has been so-called “the hallmark of productive authors,” as individuals with a greater number of self-authored articles are more likely to engage in self-citation [8]. Legitimate self-citation enables authors to build upon prior research hypotheses, replicate established methodologies, and justify the support for new studies [9]. Conversely, unethical behaviors such as excessive and unnecessary self-citation have been condemned for artificially boosting citation-based metrics and self-promotion [10]. Few studies have examined the author’s self-citation within medical literature [9, 11–14]. Moreover, researchers such as Bartneck and Kokkelmans [15] have focused on detecting manipulation of academic metrics like the h-index through self-citation analysis, underscoring the potential for self-citation to distort measures of scholarly impact and influence. Interestingly, Livas et al. (2021) [14] explored the association between author self-citation in orthodontics and factors such as author origin and gender, suggesting that sociodemographic variables may influence the propensity for self-citation among scholars. These studies collectively contribute to our understanding of author self-citation as a multifaceted phenomenon with implications that extend beyond individual disciplines.

Bibliometric investigations in dentistry have primarily focused on self-citation at the journal level, uncovering relatively low rates and indicating favorable publishing conditions and citation practices [14, 16–18]. Despite the growing literature on self-citation in various academic fields, its investigation within FO remains relatively under-explored. Given the crucial role of FO in both legal and non-legal contexts, including applications such as bite mark analysis and dental age estimation for asylum seekers, and the significant implications of flawed or biased research in this field, it is imperative to critically examine the practice of author self-citation. This study aims to fill this gap by investigating the frequency, patterns, and implications of self-citation within the contemporary FO literature, thereby promoting greater transparency, accountability, and methodological rigor in this area of research. Furthermore, the study explores self-citation trends in relation to specific article and author characteristics.

## Methods

### Data collection

This study analyzed seven prominent forensic science journals, selected based on their recognition in a recent publication [19]. The journals included *Journal of Forensic Sciences* (JFS; Impact Factor [IF]: 1.5), *Forensic Science International* (FSI; IF: 2.2), *Journal of Forensic Odonto-Stomatology* (JOFS; IF: 0.947), *Journal of Forensic and Legal Medicine* (J Forensic Leg Med; IF: 1.2), *International Journal of Legal Medicine* (IJLM; IF: 2.2), *Legal Medicine* (Leg Med; IF: 1.3), and *Egyptian Journal of Forensic Sciences* (Egypt J Forensic Sci; IF: 1.3). The analysis focused on the number of forensic odontology publications within these journals over two distinct five-year intervals: 2003–2007 and 2019–2023.

### Rationale for time frame selection

The choice of the two five-year periods, 2003–2007 and 2019–2023, for analyzing FO publications is based on several important factors. Firstly, we aim to analyze changes in self-citation patterns within FO over an extended period. The selected timeframes represent distinct phases in forensic science publishing, allowing for a comparative analysis of trends before and after notable developments in publishing practices, including the proliferation of digital databases, the implementation of open-access models, and shifts in citation behaviors. The 2003–2007 interval offers insights into FO literature during the early adoption of digital publishing, while the 2019–2023 interval captures contemporary trends influenced by the extensive use of online platforms, advanced citation tools, and enhanced international collaboration.

Secondly, these timeframes were selected to complement a recent study [19] that analyzed trends in FO publications from 2000 to 2015. The study identified a notable increase in FO research output as the field advanced, particularly in more recent years. The limited volume of FO research during the 2003–2007 period is especially relevant, highlighting the discipline’s developmental trajectory. Comparing this earlier phase with the 2019–2023 period provides valuable insights into shifts in self-citation rates and publication trends. The 2003–2007 interval serves as a baseline for FO publication activity, while the 2019–2023 timeframe represents the contemporary state of the field. Variations in publication volume between these periods reflect the growth of FO and its increasing impact on the broader forensic science literature.

### Data screening

The selection of FO publications was carried out in three stages. In the first stage, two investigators, both qualified forensic odontologists with ten years of experience (first author, NA and last author, SBB), independently accessed all journal issues from January to December from 2003 to 2007 and 2019 to 2023 through institutional subscriptions. They manually searched for original research articles, reviews, and case reports related to FO, screening the articles based on their titles and abstracts. Articles outside these categories were excluded from the analysis. In the second stage, one week after the initial screening, the selected publications underwent further manual review to confirm their relevance to FO. In cases of uncertainty, the two investigators communicated to reach a consensus on whether to include or exclude the publication. During the third stage, a more rigorous selection was applied to assess whether publications engaged in self-citation practices, using an approach similar to Livas et al. (2021) [14].

### Data extraction

The following information was extracted from each included article for analysis: the name of the journal, article title, number of authors, names of first and last authors, author rank, type of study, the topic of the study, total number of citations, number of self-citations, self-citation rate (SCR), gender, region and country of the most self-citing author, and lastly, the institutional information.

To facilitate data analysis, regions were categorized into seven groups: Asia, Africa, North America, South America, Oceania, and “others” (a combination of continents). Articles were also classified by topic, including dental age estimation, dental sex estimation, both sex and age estimation, human dental identification, bite mark analysis, forensic odontology practices, and an “Other” category, which covered areas such as dental trauma, neglect, artificial intelligence, malpractice, odontometrics, and professional liability. Additionally, articles were grouped by the number of authors: 1–3, 4–5, 6–7, and 8 or more. The author rank refers to the ranking of the authors on the number of self-citations. It is categorized as first, last, or first/ last. The first/ last refers to articles where an equal number of self-citations were observed for the first and last authors. Information regarding gender, country, and institutional status was recorded based on self-citations. In each article, the self-citations of both the first and last authors were counted to calculate the SCR, which is the percentage of an author’s self-citations relative to the total citations in the reference list. Authors with the highest SCR had their data entered into the analysis. If both authors had the same SCR, the first author’s information was used for data entry and analysis. Both FO investigators (NA & SBB) carried out the data screening. Any discrepancies were resolved through discussion until they reached a consensus.

In cases where a scientific paper indicated that the first two authors contributed equally, only the first listed author was considered in the evaluation. The collected data were then entered into a Microsoft Excel spreadsheet (Microsoft Corporation, Redmond, VA, USA) for further analysis. One month after the initial registration in the Excel spreadsheet, articles that included self-citations were reviewed to verify the accuracy of the recorded information.

### Statistical analysis

Descriptive statistical analyses were performed to explore potential associations between self-citations and various predictor variables. In addition, the selected publications were classified into two categories based on the publication periods: 2003–2007 and 2019–2023. A comparative analysis across these periods was conducted using Pearson’s chi-square test for categorical variables and the Mann-Whitney U-test for numerical data.

A negative binomial regression model was used to examine the associations between self-citations and various predictors. The categorical predictors included journal, study type, article topic, number of authors, the rank of the most self-citing author, origin, and gender, while the numerical predictor was the total number of citations. The author’s rank, origin, and gender were collected and analyzed only for articles with at least one self-citation. Consequently, the number of articles included in each univariate analysis varied, leading to different sample sizes across analyses. The distribution of the outcome variable, self-citations, was assessed using the Kolmogorov-Smirnov test, which indicated that it did not follow a Poisson distribution and showed overdispersion, justifying the use of a negative binomial regression model. Significant predictors identified in the initial univariate analysis were then entered into a multivariable negative binomial model. The significance of each predictor was assessed using likelihood ratio tests.

The box plot visualization was created in RStudio 2024.04.2 + 764 utilizing the ggplot2 package. All statistical analyses were performed using SPSS Statistics 26.0 (IBM, USA), with a significance level set at 5% (*p* < 0.05).

## Results

A total of 417 articles related to FO were identified through the search of journal issues from the 2003–2007 and 2019–2023 time periods. After a second screening phase, one editorial and one letter to the editor were excluded, resulting in 415 articles.

IJLM and the JFOS accounted for the highest number of articles, representing 20.2% and 26.0% of the total publications across both time periods. The proportions in the subsequent period were similar, with 23.4% for IJLM and 22.2% for JFOS. However, during the earlier period, the JFOS published the largest proportion of articles (38.9%) followed by the JFS (29.5%). During this period, no FO publications were identified in the Journal of Forensic Leg Med or the Egypt J Forensic Sci. A significant association was observed between the number of FO publications over time and the journal titles (*p* < 0.001) (Table 1).

**Table 1:** Distribution of articles per journal, study type, topic, and number of authors, n (%)

Category	All	2003–2007	2019–2023	*p*-value
Journal				
IJLM	84 (20.2%)	9 (9.5%)	75 (23.4%)	**< 0.001**
JFS	56 (13.5%)	28 (29.5%)	28 (8.8%)	
FSI	74 (17.8%)	16 (16.8%)	58 (18.1%)	
JFOS	108 (26.0%)	37 (38.9%)	71 (22.2%)	
J Forensic Leg Med	20 (4.8%)	0 (0%)	20 (6.3%)	
Leg. Med	43 (10.4%)	5 (5.3%)	38 (11.9%)	
Egypt. J. Forensic Sci	30 (7.2%)	0 (0%)	30 (9.4%)	
**Study type**				
Prospective	50 (12.0%)	17 (17.9%)	33 (10.3%)	0.350
Retrospective	206 (49.6%)	42 (44.2%)	164 (51.3%)	
Cross-sectional	67 (16.1%)	13 (13.7%)	54 (16.9%)	
Case report	22 (5.3%)	8 (8.4%)	14 (4.4%)	
Review or meta-analysis	53 (12.8%)	8 (8.4%)	45 (14.1%)	
Other	17 (4.1%)	7 (7.4%)	10 (3.1%)	
**Topic**				
Dental age estimation	232 (55.9%)	34 (35.8%)	198 (61.9%)	**< 0.001**
Dental sex estimation	16 (3.9%)	6 (6.3%)	10 (3.1%)	
Dental age & sex estimation	3 (0.7%)	0 (0%)	3 (0.9%)	
Identification	108 (26.0%)	35 (36.8%)	73 (22.8%)	
FO	5 (1.2%)	2 (2.1%)	3 (0.9%)	
Bitemarks	27 (6.5%)	15 (15.8%)	12 (3.8%)	
Other	24 (5.8%)	3 (3.2%)	21 (6.6%)	
**Number of authors**				
1–3	117 (28.2%)	43 (45.3%)	74 (23.1%)	**< 0.001**
4–5	135 (32.5%)	37 (38.9%)	98 (30.6%)	
6–7	103 (24.8%)	12 (12.6%)	91 (28.4%)	
8+	60 (14.5%)	3 (3.2%)	57 (17.8%)	
**Self-citation**				
Yes	237 (57.1%)	50 (52.6%)	187 (58.4%)	0.315
No	178 (42.9%)	45 (47.4%)	133 (41.6%)	

With regard to study types, the majority of articles were classified as retrospective designs (49.6%) and cross-sectional designs (16.1%). Reviews, prospective studies, and case reports represented smaller proportions, accounting for 12.8%, 12.0%, and 5.3% of the articles. The category ‘Other’ comprised 4.1% of FO publications. There was no significant difference in the proportions of FO publications across various study types in different periods (*p* = 0.350).

The most frequently addressed topics were dental age estimation (55.9%) and human dental identification (26.0%). The proportion of articles covering dental age estimation increased markedly from 2003 to 2007 to 2019–2023 (35.8% vs. 61.9%). In contrast, the most notable decline in articles was observed in human dental identification (36.8% vs. 22.8%) and bitemarks (15.8% vs. 3.8%). The observed discrepancy in the frequency of covered topics was statistically significant (*p* < 0.001).

Regarding authorship, the data revealed that 28.2% of the articles had one to three authors, 32.5% had four to five authors, 24.8% had six to seven authors, and 14.5% had eight or more authors. The number of authors contributing to forensic odontology (FO) articles exhibited a notable change over time (*p* < 0.001). Between 2003 and 2007, most articles had one to three (45.3%) or four to five (38.9%) authors. In contrast, the period between 2019 and 2023 demonstrated a shift towards a larger number of co-authors. Compared to the figures from 2003 to 2007, the proportion of articles with one to three authors decreased, while the proportion with four to seven authors increased. Additionally, the proportion of articles with eight or more authors rose from 12.6 to 17.8%.

Two hundred thirty-seven articles (57.1%) included at least one self-citation. This proportion did not change significantly over time (*p* = 0.315). The proportion of articles with self-citations was 52.6% from 2003 to 2007 and increased to 58.4% from 2019 to 2023 (Table 1).

**Fig. 1:**
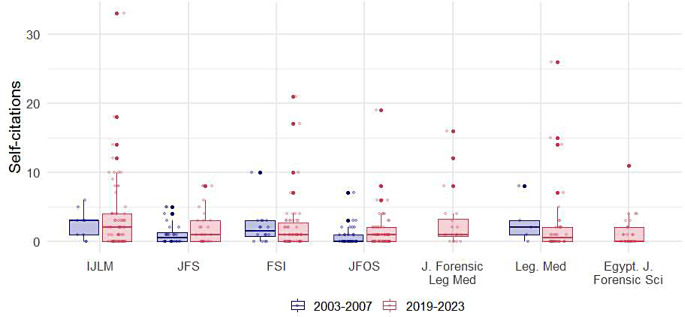
Distribution of self-citations by journal

**Fig. 2:**
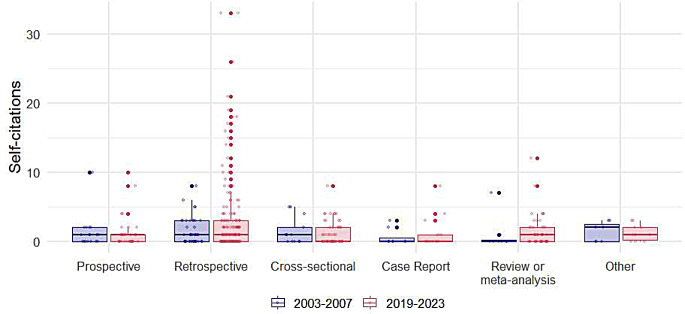
Distribution of self-citations by study type

**Fig. 3:**
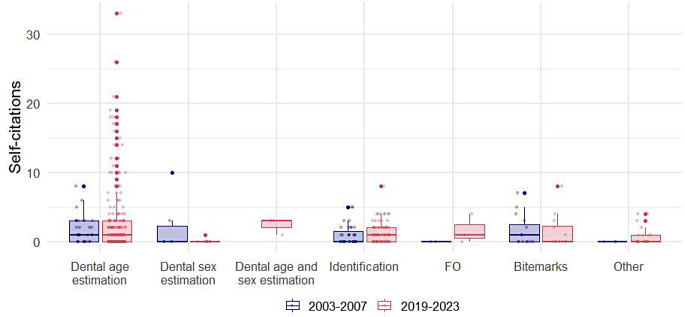
Distribution of self-citations by topic

As shown in Table 2, among the 415 selected articles, the median total number of citations was 31 (IQR: 22.5, 42), while the median number of self-citations was 2 (IQR: 1, 4). The median self-citation rate (SCR) was 7.4% (IQR: 3.8–15.7%). The median number of citations increased significantly from 21 (IQR: 13.8, 28.3) during the period from 2003 to 2007 to 33 (IQR: 26, 45) during the period from 2019 to 2023 (*p* < 0.001). Although there was no statistically significant change in the number of self-citations over time (*p* = 0.220), the SCR decreased by nearly twofold, from 11.3% (IQR: 5.4, 20.4) to 6.7% (IQR: 3.6, 13.8) (*p* = 0.007). Boxplots for self-citations per journal, study type, and topic are illustrated in Figs. 1 and 2, and 3, respectively.

**Table 2:** Continuous citation and self-citation metrics, me (C_25_, C_75_)

Variable	All	2003–2007	2019–2023	*p*-value
Number of citations	31 (22.5, 42)	21 (13.8, 28.3)	33 (26, 45)	**< 0.001**
Number of self-citations	2 (1, 4)	2 (1, 3)	2 (1, 4)	0.220
Self-citation rate (SCR), %	7.4 (3.8, 15.7)	11.3 (5.4, 20.4)	6.7 (3.6, 13.8)	**0.007**

Upon examining the author rank in articles that included self-citations, it was observed that 27.0% of these articles contained self-citations by the first author. In contrast, the last authors were more likely to self-cite, accounting for 54.0% of the articles with self-citations. Additionally, 19.0% of the articles showed self-citations from both the first and last authors. No significant differences in proportions were observed between the analyzed time periods (*p* = 0.269).

Regarding the gender of the authors, the majority of self-citers were male, comprising more than twice the proportion of female authors (71.9% vs. 28.1%). The gender distribution of authors citing their own work showed a statistically significant change over time (*p* = 0.006). Specifically, the proportion of self-citing male authors decreased from 89.7 to 67.5%, while the proportion of female authors citing their own work more than tripled, increasing from 10.3 to 32.5%.

**Fig. 4:**
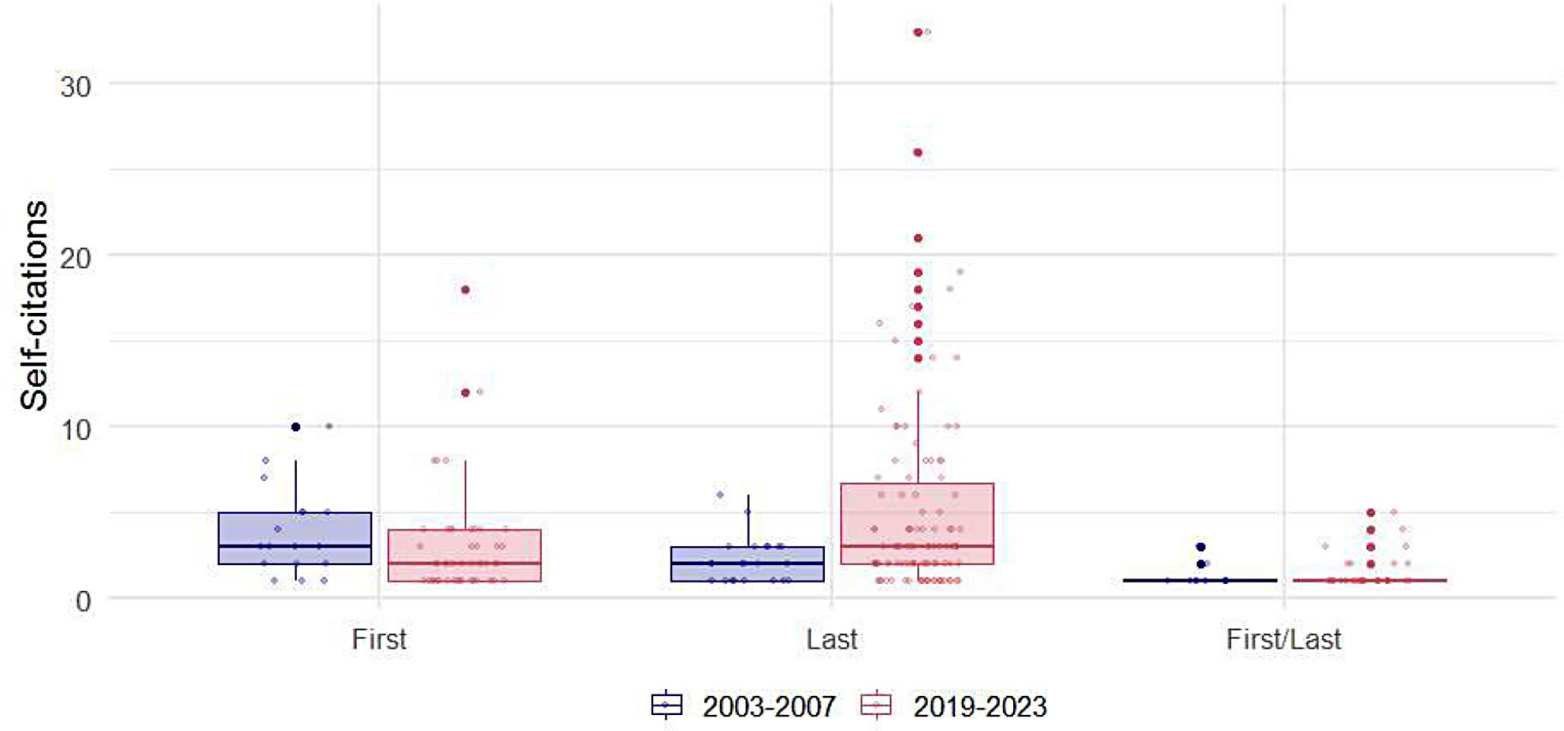
Distribution of self-citations by self-citing author’s rank

**Fig. 5:**
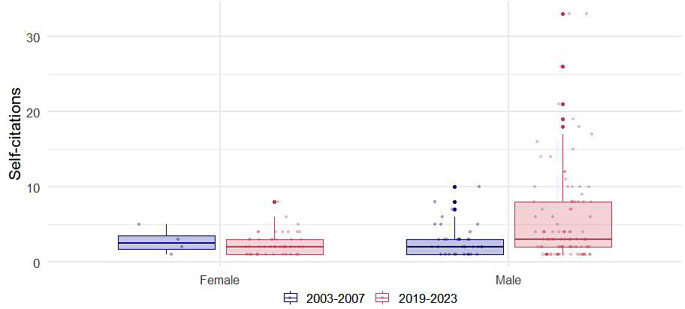
Distribution of self-citations by self-citing author’s gender

**Fig. 6:**
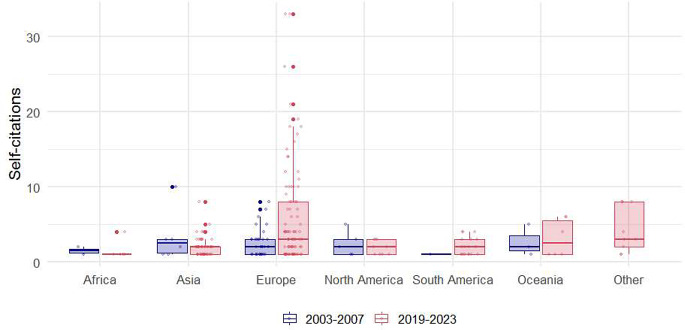
Distribution of self-citations by self-citing author’s origin

The authors of European origin were the most frequent self-citers, representing 51.1% of the total, followed by those of Asian origin at 24.5%. There was a significant change in the proportions of authors from different origins over time (*p* = 0.021). The proportion of authors of Asian origin increased by more than twofold between 2003 and 2007 and 2019 to 2023 (12.0% vs. 27.8%). The most notable increase in the proportion of self-citing authors was observed among those from South America, which saw a fourfold increase (2.0% vs. 9.6%). Conversely, the most dramatic decreases were seen in authors from Europe (66.0% vs. 47.1%) and North America (10.0% vs. 4.8%) (Table 3). Boxplots for self-citations per self-cited author’s rank, gender, and origin are illustrated in Figs. 4 and 5, and 6, respectively.

**Table 3:** Distribution of articles with at least one citation per most self-cited author rank, gender, and origin

Category	All	2003–2007	2019–2023	*p*-value
**Rank**				
First	64 (27.0%)	17 (34.0%)	47 (25.1%)	0.269
Last	128 (54.0%)	22 (44.0%)	106 (56.7%)	
First/Last	45 (19.0%)	11 (22.0%)	34 (18.2%)	
**Gender**				
Male	141 (71.9%)	35 (89.7%)	106 (67.5%)	**0.006**
Female	55 (28.1%)	4 (10.3%)	51 (32.5%)	
**Origin**				
Africa	7 (3.0%)	2 (4.0%)	5 (2.7%)	**0.021**
Asia	58 (24.5%)	6 (12.0%)	52 (27.8%)	
Europe	121 (51.1%)	33 (66.0%)	88 (47.1%)	
North America	14 (5.9%)	5 (10.0%)	9 (4.8%)	
South America	19 (8.0%)	1 (2.0%)	18 (9.6%)	
Oceania	9 (3.8%)	3 (6.0%)	6 (3.2%)	
Other	9 (3.8%)	0 (0%)	9 (4.8%)	

The application of univariable negative binomial regression, based on the likelihood ratio test, revealed significant associations between the number of self-citations and several factors: the journal (*p* < 0.001), study type (*p* < 0.001), and topic (*p* = 0.001). Additionally, significant factors included the number of authors (*p* = 0.001), the rank of the self-cited author (*p* < 0.001), their gender (*p* < 0.001), and origin (*p* < 0.001).

The analysis indicated that articles published in the IJLM had a higher likelihood of self-citations (*p* = 0.015), as did articles with fewer co-authors—specifically, those with one to three (*p* = 0.003) and four to five authors (*p* = 0.002). The first and last authors were more likely to cite their work, with the incidence rate ratio of self-citations being 3.4 times higher for the last author and 2.3 times higher for the first author than the reference group (*p* < 0.001 for both).

Moreover, male authors exhibited a twofold increased likelihood of self-citation compared to their female counterparts (*p* < 0.001). The incidence rate ratio for self-citation was significantly lower among authors from Asia, Africa, and South America (*p* = 0.045, *p* = 0.030, *p* = 0.041, respectively). Despite the model’s overall significance, no specific topic emerged as a predictor of self-citation frequency (Table 4). Although a significant association was identified in the univariable analysis, the multivariable analysis did not reveal any statistically significant associations between self-citations and the predictor variables included in the analysis.

**Table 4:** Univariate negative binomial regression results

Predictor	Univariable		
	IRR	95% CI	*p*-value
**Journal**			
IJLM	1.973	1.141, 3.411	0.015*
JFS	1.039	0.570, 1.891	0.901
FSI	1.110	0.630, 1.953	0.719
JFOS	0.875	0.497, 1.539	0.643
J Forensic Leg Med	1.353	0.688, 2.661	0.380
Leg. Med	1.598	0.864, 2.955	0.135
Egypt J. Forensic Sci	ref.**		
**Study type**			
Prospective	1.151	0.566, 2.344	0.698
Retrospective	2.229	1.195, 4.158	0.012*
Cross-sectional	1.294	0.651, 2.571	0.462
Case report	1.375	0.560, 3.373	0.487
Review or meta-analysis	1.329	0.656, 2.690	0.430
Other	ref.**		
**Topic**			
Dental age estimation	1.924	0.986, 3.755	0.055
Dental sex estimation	2.000	0.605, 6.614	0.256
Dental age & sex estimation	1.000	0.271, 3.690	1.000
Identification	0.995	0.495, 2.002	0.990
FO	1.071	0.236, 4.865	0.929
Bitemarks	1.385	0.605, 3.170	0.441
Other	ref.**		
**Number of authors**			
1–3	0.564	0.386, 0.824	0.003*
4–5	0.571	0.398, 0.820	0.002*
6–7	0.908	0.634, 1.300	0.599
8 or more	ref.**		
**Rank**			
First	2.310	1.560, 3.421	< 0.001*
Last	3.426	2.401, 4.889	< 0.001*
First/Last	ref.**		
**Gender**			
Male	2.051	1.525, 2.758	< 0.001*
Female	ref.**		
**Origin**			
Africa	0.372	0.142, 0.979	0.045*
Asia	0.506	0.274, 0.936	0.030*
Europe	1.208	0.677, 2.155	0.523
North America	0.491	0.229, 1.052	0.067
South America	0.474	0.231, 0.971	0.041*
Oceania	0.711	0.314, 1.608	0.412
Other	ref.**		

**IRR** Incidence rate ratio

* significance

** reference category

## Discussion

Bibliometric analyses of author self-citation can help identify individuals who disproportionately cite their own work, potentially skewing citation metrics in academia. Lead or first authors, often early-career researchers, may demonstrate higher self-citation rates due to the relatively recent publication of their work, limiting the time available for external citations to accumulate [20]. However, in line with previous research [11, 14], our findings indicate that the last authors exhibited higher self-citation rates than the first authors. In scientific publishing, the first and last authors are typically regarded as the primary contributors to multi-author papers [21]. Given that the last authors are frequently senior researchers holding esteemed academic positions, self-citation in this group may be more common, reflecting their extensive publication histories [2].

Our analysis reveals that author origin is a significant factor in self-citation patterns, with a higher prevalence of self-referencing among European and Asian authors. This aligns with the findings of Livas et al. [14], who also reported a markedly higher rate of self-citations among European authors. While the gender disparity in self-citation has diminished over time, decreasing from approximately nine times higher in men during the 2003–2007 period to just over two times higher in the 2019–2023 period, male authors still exhibited a self-citation frequency about three times greater than their female counterparts. A similar male dominance in self-citation has been observed in various academic fields [2, 14].

To our knowledge, this study is the first to investigate synchronous author self-citation within the FO, a subfield of forensic sciences. The only other known study examining self-citation rates in the forensic field reported an 8.9% SCR and highlighted the variability of SCRs across different journals [22]. A key strength of our study is the comprehensive inclusion of journals and the extensive timeframe for data collection. Notably, our analysis covered two broad periods (2003 to 2007 and 2019 to 2023) across seven leading forensic sciences journals. While the median number of self-citations remained constant, we observed a significant increase in the median number of citations per article, which led to a decrease in the SCR by nearly half, decreasing from 11.3% in 2003–2007 to 6.7% in 2019–2023.

It is important to acknowledge the legitimate reasons and underlying hypotheses that explain the self-citation phenomenon. In FO, several key arguments support the practice of self-citation. Researchers in FO often engage in pioneering work, such as development of techniques for dental age estimation. Self-citation allows these researchers to acknowledge their foundational contributions, which serve as crucial reference points for subsequent and future research. This practice highlights the progressive nature of scientific inquiry in the field. FO is a specialized discipline with a relatively small community of experts. In such a context, self-citation may not merely reflect self-promotion but rather serve as a necessary means of citing and validating ongoing research. This ensures that the cumulative knowledge generated by these experts is appropriately recognized and integrated into further research. Additionally, maintaining methodological consistency is crucial in FO, and researchers may self-cite to ensure accurate replication and validation of their methodologies. This practice is essential for preserving the integrity of research outcomes, particularly in forensic sciences, where precision and reliability are paramount.

Some researchers suggest that SCRs of 10–20% are perceived as normal, while rates exceeding this range are often viewed as excessive [23, 24]. However, it is important for authors to use self-citations thoughtfully and in moderation, ensuring they enhance the credibility and context of their research without overemphasizing their work [3]. When done appropriately, self-citation helps ground new researchin an established scientific context. Addressing knowledge gaps in FO requires referencing prior research to provide context and continuity. Self-citation serves as a bridge, connecting past insights with new findings, effectively addressing literature gaps, and ensuring that a solid scientific foundation supports current studies. However, self-citation rates should be kept to a reasonable level to maintain the integrity and objectivity of the research. Excessive self-citation risks introducing bias, potentially distorting the findings, and may be perceived as self-promotion rather than a legitimate scientific reference [25].

### Limitations of the study

The study has several notable limitations that warrant consideration. Firstly, the focus on only two decades of publications represents a narrow time frame, potentially limiting the ability to capture the full scope of self-citation trends over a longer period. Specifically, we only investigated the phenomenon over 2003–2007 and 2019–2023. We have not explored historically whether self-citation has been observed in previous decades since the establishment of FO as a science. Furthermore, the inclusion of specific journals for analysis may restrict the generalizability of findings, as different journals could exhibit varying self-citation practices. Additionally, the study’s reliance on a specific definition of self-citation, primarily direct citations by the same author, may overlook more nuanced forms like indirect citations or collaborations between authors. Although data on specific countries, institutional affiliations, and author names were collected, the authors of this study chose to exclude these variables from the analysis to protect the confidentiality of the authors. This decision was made due to the sensitive nature of the data and the challenges associated with conducting a quantitative analysis of the various unique and combined values that would have resulted from including such information. Additionally, revealing this information could raise ethical concerns and compromise the privacy of the authors and their institutions. To ensure the authors’ identities are protected and to avoid any potential conflicts of interest, these variables were left out of the analysis, allowing the study to focus on the primary research question.

When interpreting the results, it is important to remember that the comparison of predictor impacts should be made relative to the reference group, as the model estimates changes in self-citations in comparison to a baseline category. Therefore, the magnitude and significance of the effects should be considered in relation to the reference group, rather than in absolute terms, to fully understand the relationships between self-citations and the predictors.

This study is subject to another limitation: only the names of the first and last authors were considered and evaluated during the article screening process. It is important to acknowledge the limitation of this study, as some journals published only a very limited number of articles on FO topics within one year. Another limitation of this study is the potential impact of journals with no published issues during certain periods on the reliability of the results. Specifically, our negative binomial regression analysis included all journals in the dataset, but some had no issues published during the analyzed periods. For instance, the Egyptian Journal of Forensic Sciences had no issues between 2003 and 2007, the Journal of Forensic Sciences had no issues between 2003 and 2005, and the Journal of Forensic and Legal Medicine had no issues between 2003 and 2006. The inclusion of these journals with zero publication counts during these periods may have influenced the model’s estimates and the overall reliability of the results, potentially leading to biased or inflated estimates of publication trends. Future studies should consider addressing this limitation using alternative analytical approaches or excluding journals with zero publication counts during certain periods.

### Future directions of this research

Looking forward, there are several key areas for future research that could enhance our understanding of self-citation within FO and its broader implications. Longitudinal bibliometrics studies spanning extended periods could provide a valuable insights into how self-citation patterns evolve, highlighting emerging trends and potential shifts in research priorities. Future investigations should explore both synchronous and diachronous SCRs and patterns over extended observation periods, encompassing a broader range of forensic science journals, including both English and non-English publications, as well as journal with varying impact factors and those indexed in different databases. In addition to quantitative analyses, qualitative approaches, such as interviews or surveys with researchers, would offer deeper insights into the motivations behind self-citation practices. Further bibliometric research is recommended to comprehensively analyze the author’s self-citation practices in FO and compare them with those in other subfields of forensic science. Future directions of this research should include investigating whether the presence of research funding influences self-citation in first-author publications and whether the article is open access.

## Conclusion

Based on the literature, this study represents the first scientific work into self-citation within FO. The self-citation patterns observed among first and last authors in journals specializing in forensic sciences, which include FO publications, mirror those identified in medical specialties. Despite the limited number of articles examined and the narrow time frame of this investigation, it is noteworthy that author origin and gender show correlations with self-citation behaviors. This study lays the groundwork for future research endeavors, suggesting exploring longer time spans to further elucidate these dynamics.

## Key Points

1. Analyzed author self-citations in contemporary Forensic Odontology literature (2003–2023).
2. 57.1% of the reviewed articles included self-citations.
3. Further research needed on long-term self-citation patterns.
